# Parasite-mediated inbreeding depression in wild red deer

**DOI:** 10.1038/s41437-025-00801-w

**Published:** 2025-10-01

**Authors:** Adam Z. Hasik, Anna M. Hewett, Katie Maris, Sean J. Morris, Ali Morris, Gregory F. Albery, Josephine M. Pemberton

**Affiliations:** 1 Institute of Ecology and Evolution, University of Edinburgh, Edinburgh, UK; 2 Department of Ecology and Evolution, University of Lausanne, Lausanne, Switzerland; 3 Department of Zoology, Trinity College Dublin, Dublin, Ireland

**Keywords:** Inbreeding, Evolutionary ecology

## Abstract

Inbreeding depression is the reduction in fitness of inbred individuals relative to their more outbred counterparts. Parasitism also reduces fitness and is a route by which inbreeding depression may operate, yet the complete pathway from inbreeding to parasitism to fitness has almost never been documented in the wild. We investigated parasite-mediated inbreeding depression in a wild population of a large mammal (red deer, *Cervus elaphus*), using high-quality individual-level data on fitness in juveniles and adult females, longitudinal infection data for three gastrointestinal helminth parasites, and genomic inbreeding coefficients. We found evidence for parasite-mediated inbreeding depression via strongyle nematodes in juvenile survival, independent of direct adverse effects of inbreeding on survival and indirect effects of inbreeding on survival via birth weight. Inbreeding also reduced fitness in reproductive adults by reducing overwinter survival. Our study reveals three independent pathways by which inbreeding depresses fitness and highlights the rarely-studied route of parasitism.

## Introduction

Inbreeding depression, the reduction in fitness in the offspring of related parents, is a universal phenomenon of diploids. It is ascribed to two mechanisms: the expression of (partially recessive) deleterious alleles, generally thought to be the more important process, and loss of heterozygosity at loci with heterozygote advantage (overdominance) (Charlesworth and Willis 2009). Although we have known about inbreeding depression since Darwin (Darwin 1876), its study in natural populations with biparental inbreeding (i.e., including most animals) has been relatively slow to develop due to the requirement for both estimates of individual fitness and pedigrees or other tools for assessing individual identity-by-descent (IBD). With the advent of genomic approaches for assessing IBD, it is now clear that inbreeding depression in the wild is often more severe than has been documented in the past (Kardos et al. 2015; Kardos et al. 2016).

Inbreeding depression specifically refers to fitness. Fitness is a hard-to-measure, complex trait which is the outcome of many aspects of an individual’s development and physiology, and these in turn are determined by a combination of environmental and genetic sources of variation. Traits such as body size are often positively related to fitness components such as survival and are easier to measure than fitness or its components, so many studies of inbreeding depression in the wild focus on such fitness-related traits. However, inbreeding depression (*sensu stricto*) is likely to be expressed via multiple different pathways and at present we have little idea of their nature and relative importance in inbreeding depression.

Parasitism is a strong selective force in natural populations (Hasik and Siepielski 2022a), having led to the continued polymorphism in the mammal major histocompatibility complex (MHC, Ebert and Fields 2020; Eizaguirre et al. 2012; Haldane 1949; Klein et al. 1994) and the maintenance of sexual reproduction among eukaryotes (Auld et al. 2016; Hamilton et al. 1990), and parasite-mediated effects have the potential to scale up to the macroevolutionary level (Hasik et al. 2025b). A strong theme within host-parasite coevolution research is the concept that parasites can select for host genetic diversity by increasing the number of host genotypes, preventing parasite adaptation to a common genotype (the Red Queen Hypothesis, Auld et al. 2016; Hamilton et al. 1990). At the individual-level, host genetic diversity, i.e. heterozygosity can in principle affect susceptibility to parasite infection via two routes. Homozygosity at specific parasite defense loci may reduce fitness if two different alleles confer better parasite resistance than one, i.e. there is overdominance. Alternatively, genome-wide homozygosity can lead to increased susceptibility to parasite infection (Budischak et al. 2023). The fitness costs associated with reduced individual-level genetic diversity due to inbreeding (i.e., inbreeding depression) are yet another path by which the selective forces of parasitism could impact a population’s genetic diversity.

The relationship between inbreeding and parasitism in natural populations has been researched in a number of natural populations, though often with imprecise measurement of inbreeding. Studies of Galápagos hawks (Whiteman et al. 2006), mountain white-crowned sparrows (MacDougall-Shackleton et al. 2005), and California sea lions (Acevedo-Whitehouse et al. 2006) have found that parasitism increases with estimates of individual inbreeding. However, these examples only considered the relationships between inbreeding and parasitism. Further, these studies also suffer from the fact that they used relatively crude measures of individual inbreeding. For example, the relationship between heterozygosity at a few microsatellites and inbreeding coefficients from a deep pedigree is often weak (Balloux et al. 2004; Slate et al. 2004). With time, there has been an expansion in the size and completeness of genetically-supported wild pedigrees, enabling more precise estimation of pedigree inbreeding coefficients (Sardell et al. 2010). This approach has itself been largely supplanted by our ability to genotype thousands of genome-wide markers (Kardos et al. 2015; Kardos et al. 2016). Genomic measures of individual inbreeding, such as genome-wide homozygosity (Hoffman et al. 2014), metrics derived from the genomic relationship matrix (Yang et al. 2011), and runs of homozygosity (McQuillan et al. 2008) now offer even more precise estimates of individual inbreeding than pedigree inbreeding coefficients. This is because they account for inbreeding due to ancestors that pedigrees do not include, and also because whereas pedigree inbreeding coefficients are the mean expectation of IBD, genomic tools generate the realized IBD that results from the random events of Mendelian segregation and recombination. In consequence, inbreeding metrics derived from genome-wide SNP markers generally find stronger evidence of inbreeding depression than all previous methods (Bérénos et al. 2016; Hoffman et al. 2014; Huisman et al. 2016; Kardos et al. 2015; Kardos et al. 2016). For example, a study using ~100 SNP loci in orca (*Orcinus orca*) did not detect convincing evidence of inbreeding depression (Ford et al. 2018), but a study of the same population using a whole-genome approach found strong evidence for severe inbreeding depression (Kardos et al. 2023).

A complete understanding of parasite-mediated inbreeding depression in wild populations requires three individual-based components: accurate estimates of the level of inbreeding (estimated from a pedigree or genomic information), parasitism, and, crucially, fitness. Linking these three components is key, as it may uncover the effects of inbreeding on parasitism, along with any subsequent fitness loss. To date, only one study has simultaneously investigated all three components to study parasite-mediated inbreeding depression, finding that hosts that were homozygous at a panel of microsatellites had more parasites and lower overwinter survival, and that parasitism reduced overwinter survival in Soay sheep (Coltman et al. 1999). These results suggest that parasitism is a route by which inbreeding depression is expressed, but more evidence from other wild host-parasite systems is sorely needed to understand the generality of this pattern.

Our goal for this paper was to test for parasite-mediated inbreeding depression in a wild population. We investigated the links between individual-level inbreeding, parasitism by gastrointestinal helminths, and fitness through spatially-explicit analyses and structural equation models using data across two age classes from an exceptionally well-characterized study system of red deer (*Cervus elaphus*). We focused our analyses on juveniles and adult females, as parasite-mediated effects on survival in this system are apparent in both groups (Acerini et al. 2022; Albery et al. 2024; Albery et al. 2021a), and there is also evidence for spatial variation in inbreeding depression manifesting through birth weight, juvenile survival, and female lifetime breeding success (Hewett et al. 2025; Hewett et al. 2024). Linking these relationships in a single study allows us to establish what role, if any, parasites play in these relationships.

## Materials and methods

### Study system

We collected data from a focal host population of red deer on the north block of the Isle of Rum, Scotland (57°N,6°20’W). The study area runs ~4 km north to south and ~3 km east to west with a total area of ~12.7 km^2^. The population of deer within the study area is wild, unmanaged, and free from both predation and hunting. ~90% of calves born in the study area are caught soon after birth and weighed, permanently marked, and sampled for genetic analysis; individuals unsampled at this stage are recognized from natural features and are often sampled later via darting, cast antlers, or *post mortem*.

While the deer have been studied in some capacity since 1972 (Pemberton et al. 2022), data on the helminth parasite burden of the population has been non-invasively collected since 2016 by collecting fecal samples three times a year in April (Spring), August (Summer), and November (Autumn). Briefly, observers note individually recognized deer defecating from a distance and collect the fecal samples without disturbing the deer. Samples are then placed into plastic bags to keep the samples as anaerobic as possible and refrigerated at 4 °C to prevent hatching or development of parasite propagules, with subsequent parasitological examination being conducted within three weeks in the case of strongyles (Albery 2020). Detailed methods can be found in Albery (2020).

For our analysis of parasitism we focused on three of the most common parasite taxa infecting the red deer: strongyle nematodes (hereafter “strongyles”, a mix of different species with indistinguishable eggs), liver fluke (*Fasciola hepatica*), and tissue worms (*Elaphostrongylus cervi*). Strongyles have a direct lifecycle in which infective stages contaminate vegetation via fecal pellets and are subsequently consumed by a new host (Taylor et al. 2016). *F. hepatica* (Taylor et al. 2016) and *E. cervi* (Mason 1989) both have indirect lifecycles involving a snail intermediate host (the dwarf pond snail *Galba truncatula* and a number of terrestrial snails and slugs, respectively). After infecting and emerging from their intermediate hosts, larval *F. hepatica* contaminate vegetation near water bodies which is consumed by the deer final host. In contrast, deer become infected with *E. cervi* by consuming the intermediate snail host itself. While strongyle infections develop quickly such that calves excrete eggs within 2–3 months of birth, *F. hepatica* and *E. cervi* have longer prepatent periods, resulting in low prevalences of *F. hepatica* and *E. cervi* for juveniles relative to adults (Albery et al. 2018; Gajadhar et al. 1994; Hasik et al. 2025a) (Fig. S1).

Individual inbreeding was estimated using the runs of homozygosity (ROH)-based inbreeding coefficient F_ROH_ (defined as the proportion of the genome in runs of homozygosity), which captures realized IBD (McQuillan et al. 2008). All sampled deer have been genotyped on the Illumina cervine 50 K Single Nucleotide Polymorphism (SNP) and after QC 37,396 SNPs were used to search for ROHs of minimum length 2.5 Mb in PLINK v2.0 using the physical locations of SNPs from the red deer genome assembly mCerEla1.1 (Pemberton et al. 2021). For details on QC and ROH searching see Hewett et al. (2023). With these conditions, mean F_ROH_ at birth in the Rum deer is 0.06 and ranges from 0–0.35 (Hewett et al. 2023) (Fig. S2).

In juveniles, we focused on two fitness-associated measures: birth weight and overwinter survival. Birth weight is adjusted from capture weight to account for postnatal growth (assuming an increase in weight of 0.01696 g/h). Overwinter survival is a binary response, with 1 for calves/yearlings that lived through the winter into the start of the next “deer year”, a period running from May 1 – June 30, and 0 for those calves/yearlings that died before the start of the next deer year. These data included all calves and yearlings that we had parasitism and inbreeding data for. Individuals that were shot as juveniles when they roamed outside of the study area were removed from the analysis, as we were only interested in natural mortality.

For female fitness we focused on overwinter survival and fecundity. Overwinter survival was calculated as above, while fecundity was calculated as a binary response, with 1 for females that produced a calf in the next deer year, and 0 for those females that did not produce a calf in the next deer year. As with juveniles, females that were shot when they ventured outside of the study area were removed from the analysis.

### Statistical analysis

Parasite fecal egg counts (FECs) and F_ROH_ values varied across space in both the juvenile and adult datasets (Fig. S3). Confirming results from previous studies of this system (Albery et al. 2019; Hasik et al. 2025a) we found that strongyle and *E. cervi* FECs in both datasets were more abundant in the north of the study area, while *F. hepatica* was more abundant towards the middle of the study area in the adult females (Fig. S3). Relatively high values of F_ROH_ in the juveniles were found in multiple locations throughout the study area, while they tended to be concentrated in the north for the adult females (Fig. S3). To control for this spatial autocorrelation, we used Integrated Nested Laplace Approximation (INLA) models for all analyses. INLA models are a deterministic Bayesian approach which allow for the quantification of spatial effects and have been increasingly used for spatial analyses (Albery et al. 2019; Albery et al. 2020; Zuur et al. 2017). To calculate annual centroids for each individual we used the census data, where individuals’ identities and locations (to the nearest 100 m) were recorded. We then calculated the annual centroid using a previously described pipeline for this population (Albery et al. 2022; Albery et al. 2021b) using all observations of each individual in each year. This approach uses a kernel density estimator, taking individuals’ annual centroids and fitting a two-dimensional smooth to the distribution of the data, producing a two-dimensional spatial distribution of the population. We fit all models in R version 4.2.2 (2025) using the R-INLA package (Martins et al. 2013; Rue et al. 2009).

### Model construction

We constructed separate models for each of the three parasites using parasite-specific datasets. For the juveniles the strongyle dataset included all calves and yearlings for which we had strongyle data (*n* = 1068 records from *n* = 348 deer). The *F. hepatica* dataset included all calves and yearlings for which we had liver fluke data (*n* = 655 records from *n* = 301 deer), excluding data collected from calves in their first summer as *F. hepatica* are prepatent this early in a calf’s life (Albery et al. 2018; Gajadhar et al. 1994). Finally, the *E. cervi* dataset included all calves and yearlings for which we had tissue worm data (*n* = 419 records from *n* = 213 deer), excluding data collected from calves in their first summer and autumn as *E. cervi* have an even longer prepatent period (Albery et al. 2018; Gajadhar et al. 1994). For adult females the strongyle dataset included *n* = 1698 records from *n* = 170 deer, the *F. hepatica* dataset included *n* = 1393 records from *n* = 168 deer, and the *E. cervi* dataset included *n* = 1313 records from *n* = 163 deer.

To investigate links between inbreeding and parasitism, inbreeding and fitness, and parasitism and fitness, we utilized path analyses with the D-separation method (Albery et al. 2021a), where some variables appear as both fixed effects and response variables (Shipley 2009). While path analyses are a useful tool and allow for tests of hypothesized, “causal” relationships using large amounts of observational data (Albery et al. 2021a; Hasik et al. 2024; Hasik and Siepielski 2022b), such models estimate correlational relationships and cannot show causality (Shipley 2009). Combining multiple models in this way allows for identification of variables/mediating factors that are driving the overall relationship (Albery et al. 2021a). Specifically, we constructed directed acyclic graphs (DAGs) for each parasite and age class to test if and how a given parasite mediated inbreeding depression in this system.

For juveniles, each DAG was composed of three individual models quantifying the relationships between inbreeding, parasitism, and fitness. See Fig. S4 for a graphical representation of these hypothesized relationships. For the first model we used individual-level infection data (fecal egg counts on the log scale, lEPG) as our response variable with F_ROH_, age category (calf or yearling, accounting for age-category-specific differences (Albery et al. 2018; Hasik et al. 2025a)), sex (accounting for sex-specific differences (Hasik et al. 2025a)), birth weight (in kilograms), and birth day (Julian day, accounting for the fact that individuals born earlier have higher survival (Clutton-Brock et al. 1982)) as fixed effects in a Gaussian mixed-effect model with year (categorical), maternal ID, and individual ID as random effects to account for the correlational structure of the data, maternal effects, and repeated measures respectively. Importantly for this model we mean-centered lEPG values by season to limit collinearity with other fixed effects, thus controlling for seasonal variation in parasite load (Albery et al. 2018; Hasik et al. 2025a) without including season as a term in our models. For the second model we used birth weight as the response variable with F_ROH_, sex, and birth day as fixed effects in a Gaussian mixed-effect model with year, maternal ID, and individual ID as random effects. For the third model we used overwinter survival as the response variable with F_ROH_, birth weight, age category, sex, birth day, and lEPG as fixed effects in a mixed-effect logistic regression with year, maternal ID, and individual ID as random effects.

For the adult females, each DAG was similarly composed of three individual models using different model structures (see Fig. S4). For the first model we used lEPG as our response variable with F_ROH_, age (a continuous variable, accounting for age-specific differences in parasite load (Albery et al. 2024)), and reproductive status in the current year (none: did not have a calf, summer: had a calf but it died over the summer, or winter: had a calf and raised the calf up to or through winter, accounting for known relationships between reproductive investment and parasitism (Albery et al. 2021a; Hasik et al. 2025a)) as fixed effects in a Gaussian mixed-effect model with year (categorical) and individual ID as random effects to account for the correlational structure of the data and repeated measures, respectively. We again mean-centered lEPG values by season. For the second model we used overwinter survival as the response variable with F_ROH_, age, lEPG, and reproductive status in the current year as fixed effects in a mixed-effect logistic regression model with year and individual ID as random effects. For the third model we used fecundity (binary: 1 for females that had a calf in the subsequent year and 0 for females that did not) as the response variable in a mixed-effect logistic regression with F_ROH_, age, lEPG, and reproductive status in the current year as fixed effects with year and individual ID as random effects.

Connecting these models together using DAGs allows us to investigate the hypothesized causal relationships between inbreeding, parasitism, and fitness, identifying the routes by which inbreeding depression manifests within this population. We scaled all continuous variables to have a mean of 0 and SD of 1. Though each of the models described above included important covariates such as age category, sex, and reproductive status due to their known relationships with parasitism in this system, we only present results for the relationships between inbreeding, parasitism, and fitness. The full model results can be found in the Supplemental Materials (Figs. S5, 6).

## Results

### Inbreeding depresses fitness via multiple, independent pathways

**Fig. 1:**
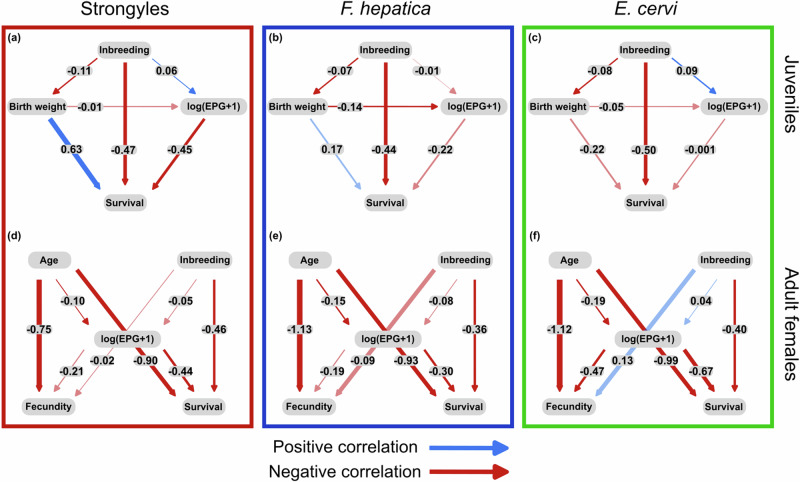
DAGs denoting the relationships between inbreeding, parasitism, and fitness in red deer. Panels denote the results for the (**a**) strongyle, **b** *F. hepatica*, and **c** *E. cervi* juvenile datasets as well as the relationships between age, inbreeding, parasitism, and fitness for the (**d**) strongyle, **e** *F. hepatica*, and **f** *E. cervi* datasets in adult female red deer. Solid lines represent standardized path coefficients of each predictor, which can be interpreted as effect sizes. Blue lines denote positive relationships, while red denote negative relationships. Significance of the effect size is denoted by opaque lines, while non-significant effect sizes are represented by faded lines. Thickness of the lines is proportional to the strength of the effects.

Our analyses revealed three routes by which inbreeding depression manifests within juveniles in the population. First, we found that F_ROH_ was negatively associated with birth weight (Fig. 1a). Given that birth weight was strongly positively associated with survival in the strongyle dataset (Fig. 1a), this indicates that inbreeding has an indirect negative effect on survival via birth weight. Second, F_ROH_ was strongly and negatively associated with overwinter survival in all three datasets (Fig. 1), confirming previous findings of inbreeding depression in this population (Hewett et al. 2024; Huisman et al. 2016; Walling et al. 2011) and providing a direct pathway by which inbreeding depression manifests (perhaps through other, unmeasured physiological variables, see the *Discussion* for more detail). The third and final route by which inbreeding depression manifests provided evidence for parasite-mediated inbreeding depression in the strongyle dataset. That is, F_ROH_ was weakly positively associated with strongyle lEPG, and lEPG was strongly and negatively associated with overwinter survival (Fig. 1a). Note that these three routes by which inbreeding depression manifests (indirect negative relationship with survival via birth weight, direct negative relationship with survival, and indirect negative relationship via strongyle lEPG) are independent of one another. lEPG was also weakly and positively associated with F_ROH_ in the *E. cervi* dataset, though there were no relationships between lEPG and survival in the *F. hepatica* or *E. cervi* datasets (Fig. 1b, c).

**Fig. 2:**
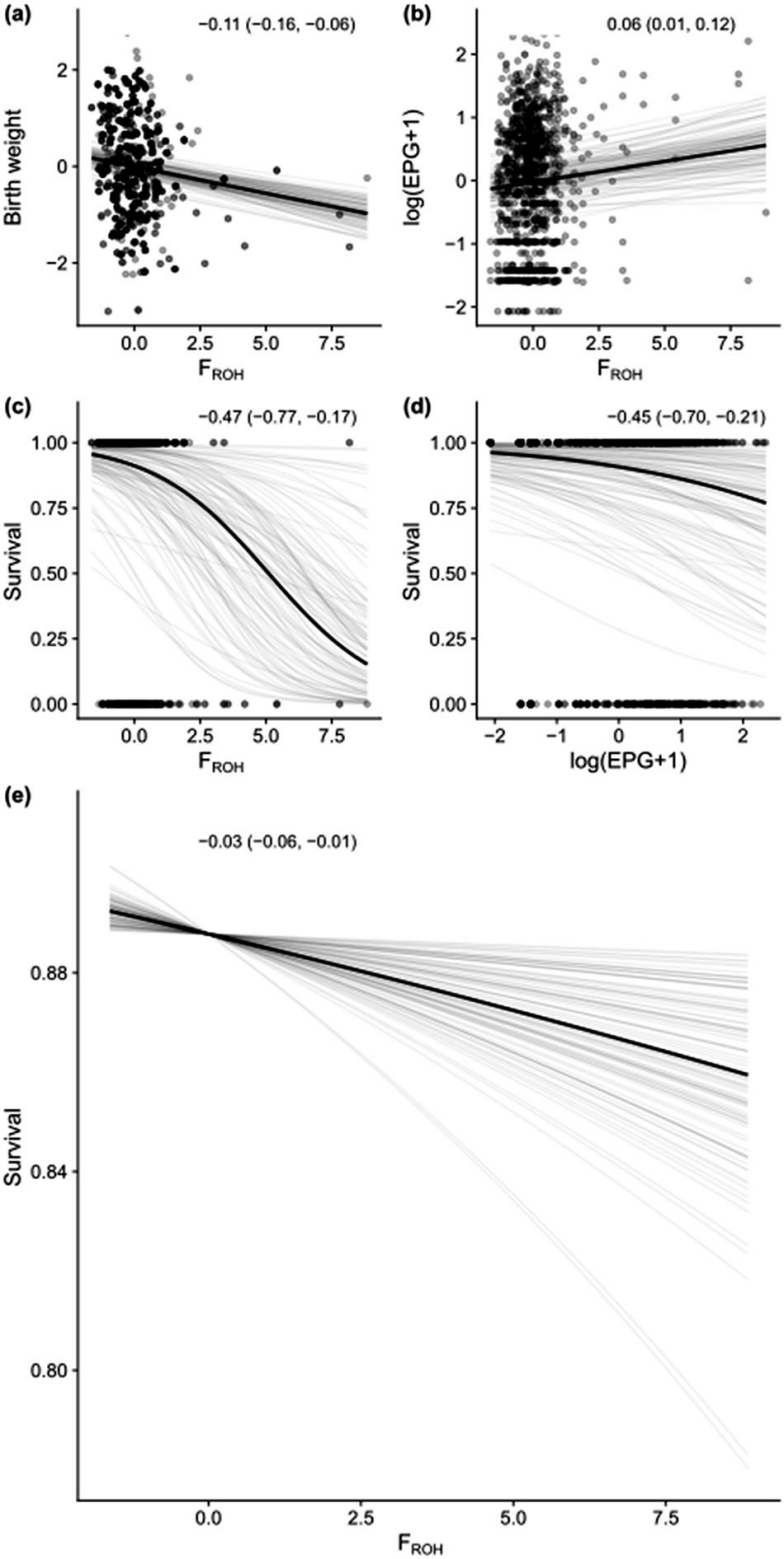
Representation of the direct relationships between inbreeding, strongyle parasitism, and survival in the juvenile stronygle data. Panels denote inbreeding and birth weight (**a**), inbreeding and strongyle parasitism (**b**), inbreeding and overwinter survival (**c**), parasitism and overwinter survival (**d**), and the indirect, strongyle-mediated relationship between inbreeding and overwinter survival (**e**). The x-axes denote standardized values for individual F_ROH_ (**a**, **b**, **c**, **e**) or individual lEPG (**d**), with birth weight (**a**), lEPG (**b**), or overwinter survival (**c**–**e**) on the y-axes. The untransformed F_ROH_ values range from 0.02–0.28 for juveniles. Results are taken from the individual models analyzing the juvenile dataset. The dark black line represents the mean of the posterior distribution for the standardized model estimates, the light grey lines are 100 random draws from the posterior to represent uncertainty. Points denote individual samples, with transparency to allow for visualization of overplotting. The inset text in each panel represents the standardized beta coefficients and associated 95% credible intervals from each regression, which can be interpreted as effect sizes.

To better visualize these independent paths between inbreeding and juvenile survival in the strongyle dataset, we plotted the direct relationships between F_ROH_ and birth weight, F_ROH_ and strongyle lEPG, F_ROH_ and overwinter survival, strongyle lEPG and overwinter survival, as well as the indirect relationship between F_ROH_ and overwinter survival mediated by strongyles (Fig. 2). Note that Fig. 2 displays standardized values for F_ROH_, the untransformed F_ROH_ values in our sample of calves range from 0.02–0.28. Birth weight declined by ~17% across the range of F_ROH_ (Fig. 2a), while strongyle lEPG increased by ~20% (Fig. 2b). Overwinter survival was reduced by ~83% and ~19% across the ranges of F_ROH_ (Fig. 2c) and strongyle lEPG (Fig. 2d), respectively. Taken together, the indirect, strongyle-mediated effect of F_ROH_ reduced overwinter survival by ~3% across the range of F_ROH_ (Fig. 2e).

### Inbreeding depression in adult female survival and fecundity

Our analysis of adult females revealed that the deleterious effects of inbreeding persist beyond the juvenile stage. Specifically, we found that inbreeding moderately reduced overwinter survival in all three datasets, though we did not find any relationships between F_ROH_ and lEPG in any of the datasets (Fig. 1d–f). We also directly linked parasitism and fitness, finding moderate, negative associations between lEPG and survival in the strongyle and *F. hepatica* datasets, in addition to a large negative relationship between lEPG and survival in the *E. cervi* dataset. lEPG was also associated with moderate reductions in fecundity in the *E. cervi* dataset (Fig. 1f). Age was associated with weak declines in lEPG for all three parasites, yet was simultaneously associated with very strong declines in survival and especially fecundity (Fig. 1).

Though it was not significant, we found that more inbred females had fewer strongyles on average (Fig. 1d). Inbreeding increased strongyle parasitism in juveniles, with such increased parasitism resulting in increased juvenile mortality (Fig. 1a). These conflicting patterns across age classes suggest that the combined effects of inbreeding and parasitism may be removing highly inbred as well as highly parasitized individuals via within-generation purging, thus explaining the lack of a pattern in adults. Indeed, the extreme values of both F_ROH_ and strongyle lEPG values are greatly reduced in adult females compared to that of juveniles (Fig. S7).

## Discussion

Identifying the mechanisms by which inbreeding depression manifests is a key first step in moving beyond a phenomenological understanding of the consequences of inbreeding, and crucial to this is understanding if and how parasites mediate inbreeding depression in wild populations. Here, using data from an exceptionally well-characterized ungulate system we linked the deleterious effects of inbreeding to parasitism, and then connected both of these negative factors to host survival in juveniles and adult females. We found that more inbred juvenile deer experienced an increased burden of strongyle nematodes, and increases in strongyle lEPG resulted in decreased overwinter survival – separately from two independent negative correlations between inbreeding and overwinter survival (one direct relationship and one indirect relationship via birth weight). These findings not only support previous findings in this system (Acerini et al. 2022; Hewett et al. 2024; Huisman et al. 2016; Walling et al. 2011), but also serve as only the second example of parasite-mediated inbreeding depression in a wild population.

The only other study to explicitly link inbreeding to parasite burden, with a subsequent link through to fitness, was Coltman et al. (1999). Like our study, Coltman et al. leveraged data from a long-term study of a wild ungulate, Soay sheep (*Ovis aries*). Similar to our results, they found that individual-level inbreeding resulted in increased strongyle burden, with strongyles being negatively associated with overwinter survival in both adults and juveniles (Coltman et al. 1999). A key advance between Coltman et al. and ours is the use of a genome-wide measure of inbreeding, F_ROH_. Coltman et al. use heterozygosity from 14 microsatellite loci, which is an imprecise representation of genome-wide IBD.

Despite finding strong, negative associations between strongyle lEPG and juvenile survival, we did not find a relationship between this measure of fitness and parasitism by *F. hepatica* or *E. cervi* FECs and survival. Previous work has shown that increasing counts of both strongyles and liver fluke were associated with reduced survival in juvenile red deer (Acerini et al. 2022), so it is interesting that we did not find a link with *F. hepatica*. The cumulative nature of our analyses may provide an explanation. Specifically, Acerini et al. (2022) found that only summer *F. hepatica* counts in yearlings were negatively associated with reduced overwinter survival. Here, we had more years of data (thus a larger sample size) and included all parasite counts from a year and analyzed calves and juveniles together to gain a more general understanding of the relationships between parasitism and inbreeding and not overfit our models, thus we may have lost some of the nuance of such season- and age-class-specific tests.

The strength of the direct relationship between strongyles and survival is similar to that of the direct relationship between inbreeding and survival, and these relationships are independent of one another. Further, we showed that F_ROH_ was negatively associated with birth weight in the strongyle dataset, mirroring the finding of Hewett et al. (2024). However, the remaining and strong negative relationship between F_ROH_ and survival points to additional, untested routes. That is, it could be that inbreeding has negative effects on other fitness-associated variables, and these factors provide further mechanisms by which inbreeding depression operates within this system. For example, inbreeding experiments with *Drosophila simulans* showed that inbreeding reduced development time (Wright et al. 2007), and early development is a key predictor of juvenile survival in this population of red deer (Clutton-Brock et al. 1987a; Kruuk et al. 1999). Birth weight, birth day, and parasitism were already included in our analyses, but other aspects of early development may have been negatively impacted by inbreeding and resulted in the observed, parasite-independent increase in overwinter mortality among inbred juveniles. In the red deer on Rum, such effects of early development can extend beyond the juvenile stage to affect adult reproduction and size (Albon et al. 1987). Our finding that adult female survival decreased with inbreeding, independent of the effects of age and parasitism, confirms previous findings that the effects of inbreeding continue past the juvenile stage (Hewett et al. 2024; Huisman et al. 2016).

This study has implications for the conservation of endangered populations. It has long been recognized that lack of genetic variation and/or the fixation of deleterious recessive alleles may make populations susceptible to disease. Two examples where this link has been suggested in the past are a cheetah (*Acinonyx jubatus*) colony that was decimated by a coronavirus in Oregon in 1983 (O’Brien et al. 1985) and the extreme susceptibility of Père David’s deer (*Elaphurus davidianus*) to malignant catarrhal fever when imported to New Zealand (Orr and Mackintosh, 1988). This link is supported by laboratory experiments. For example, extremely inbred (i.e., pedigree inbreeding coefficients of ~0.25–1) *D. melanogaster* were more susceptible to infection by *Serratia marcesans* bacteria and this increased susceptibility was due to reduced resistance to infection in more inbred individuals (Spielman et al. 2004). The red deer of Rum, with mean F_ROH_ = 0.06, are not especially inbred compared with some of the other species of conservation concern which have now been assessed by F_ROH_. Although the depth of genomic information and minimum ROH calls complicate comparison, populations of orca (Kardos et al. 2023), tigers (*Panthera tigris*) (Khan et al. 2021), Eurasian lynx (*Lynx lynx*) (Abascal et al. 2016), Scandinavian wolves (*Canis lupus*) (Kardos et al. 2017), and European ibex (*Capra ibex*) (Grossen et al. 2020) have all been recorded with higher mean F_ROH_ values than the Rum red deer. If we are able to identify an infection route for inbreeding depression in the Rum red deer, it seems likely such effects exist in these other populations and may be even stronger. Future study of parasite-mediated inbreeding depression using methods similar to ours offer a non-invasive way to quantify how vulnerable wild populations are to disease risk.

An outstanding issue in conservation genetics is the extent to which inbreeding depression, here documented in juvenile survival and female fecundity, affects population dynamics and persistence. Key to this is whether the selection associated with inbreeding is hard or soft (Kardos et al. 2024). Hard selection is expected to affect population growth as the effects of selection are additive, with selection affecting *how many* members of the population survive and reproduce, while soft selection is compensatory and affects *which* members of the population survive and reproduce (Bell et al. 2021; Wallace, 1975). Soft selection is commonly associated with density dependence, and in the deer study population there is ample evidence of density dependence. The population was released from culling in 1974 and has been at carrying capacity for many years, with a relatively stable population of females aged 1 year or older of between 150 and 200 since 1980 (Pemberton et al. 2022). Both juvenile survival and female fecundity are negatively associated with density (Clutton-Brock et al. 1987b; Coulson et al. 2000). Inbreeding depression is therefore unlikely to affect the red deer study population size on the scale of decades. This is in sharp contrast to the example of Alpine ibex populations reintroduced to depopulated environments in Switzerland, a scenario in which some hard selection is likely, where population growth is negatively associated with inbreeding (Bozzuto et al. 2019). In a hard selection scenario, the inbreeding depression documented here in red deer may well have a similar effect.

In conclusion, our study has found clear evidence for parasite-mediated inbreeding depression. Parasitism is a ubiquitous species interaction affecting host fitness, host species interactions, and host evolution across the Tree of Life (Betts et al. 2018; Buckling and Hodgson 2007; Hasik et al. 2023; Hasik and Siepielski 2022a). Through the use of high-quality, individual-level data on variation in parasitism and inbreeding, our study has identified parasites as a mechanism by which inbreeding depression manifests in wild populations. While there is more work needed to understand parasite-mediated inbreeding depression, this is a crucial first step. Genomic estimators of inbreeding are more available now than ever, and observational approaches are less invasive, which is important for endangered or threatened populations. Approaches like those utilized in this study could therefore be helpful for better understanding inbreeding depression in the wild. Additionally, more studies of other host-parasite systems are needed to increase our knowledge of if and how a common and widespread species interaction acts to drive inbreeding depression in wild populations of hosts in natural settings, as we know little about the physiological mechanisms inbreeding depression acts through, and the idea that selection by parasites might be involved is very much under-studied. Such studies could be useful and provide crucial information to conservation managers and others working with small and threatened populations that are unable to conduct more invasive sampling or manipulations.

## Supplementary information

Figures S1-S7

## Data Availability

All data and code used in this study are available on DataDryad at the following link: http://datadryad.org/stash/share/g0OA-rS950aPULMjoMqLPtaRyfDi_rbPZ-fM6RHfHRc.
